# The assessment of minimal residual disease versus that of somatic mutations for predicting the outcome of acute myeloid leukemia patients

**DOI:** 10.1186/s12935-019-0807-0

**Published:** 2019-04-04

**Authors:** Serena Salehzadeh, Francesca Guerrini, Umberto Pizzano, Susanna Grassi, Elena Ciabatti, Lorenzo Iovino, Gabriele Buda, Francesco Caracciolo, Edoardo Benedetti, Enrico Orciuolo, Matteo Pelosini, Giovanni Consani, Giovanni Carulli, Maria Rita Metelli, Francesca Martini, Francesco Mazziotta, Elisa Mazzantini, Pietro Rossi, Rita Tavarozzi, Federica Ricci, Mario Petrini, Sara Galimberti

**Affiliations:** 1 Department of Clinical and Experimental Medicine, Section of Hematology, University of Pisa, Pisa, Italy; 2 GeNOMEC School of Doctorate, University of Siena, Siena, Italy; 3 Molecular Laboratory of Hematology, AOUP, Pisa, Italy; 4 University of Rome Tor Vergata, Rome, Italy; 5 Ospedale S. Chiara, UO Ematologia, Via Roma, 67, 56126 Pisa, Italy

**Keywords:** AML, *FLT3*, *NPM1*, *WT1*, *ASXL1*, *TP53*, *IDH*, *RUNX1*, Additional mutations, AML outcome

## Abstract

**Background:**

In addition to morphological and cytogenetic features, acute myeloid leukemias are characterized by mutations that can be used for target-therapy; also the minimal/measurable residual disease (MRD) could be an important prognostic factor. The purpose of this retrospective study was to investigate if somatic mutations could represent an additional prognostic value in respect of MRD alone.

**Method:**

At baseline, 98 patients were tested for *NPM1*, *FLT3*, and for *WT1* expression; 31 for *ASXL1*, *TET2*, *IDH1*, *IDH2*, *N*-*RAS*, *WT1*, *c*-*KIT*, *RUNX1*, and *DNMT3A*. The same genes have been also tested after induction and consolidation.

**Results:**

Overall, 60.2% of our patients resulted mutated: 24.5% carried mutations of *FLT3*-*ITD*, 38.7% of *NPM1*, 48.4% of *c*-*KIT*, 25.8% of *N*-*RAS* and 19.3% of *IDH2*. The probability of achieving a complete response (CR) was higher for younger patients, with low ELN risk score, *NPM1*-mutated, with low *WT1* levels, and without *FLT3*. The presence of additional mutations represented a poor predictive factor: only 19% of these cases achieved CR in comparison to 43% of subjects without any of it. Concerning survival, it was conditioned by a lower ELN risk score, younger age, reduction > 1 log of the *NPM1* mutational burden, disappearance of *FLT3* mutations and lower *WT1* expression. Regarding the role of the additional mutations, they impaired the outcome of 20% of the already MRD-negative patients. Concerning the possibility of predicting relapse, we observed an increase of the *NPM1* mutational burden at the time-point immediately preceding the relapse (about 2 months earlier) in 50% of subjects. Similarly concerning *WT1*, an increase of its expression anticipated disease recurrence in 64% of cases.

**Conclusions:**

We demonstrated that additional somatic mutations are able to impair outcome of the already MRD-negative subjects. About MRD, we suggest a prognostic role also for the *WT1* expression. Finally, we considered as relevant the assessment of *NPM1* quantity clearance instead of the presence/absence of mutations alone. Still remains in doubt the utility in terms of long-term prognosis of a baseline more complex mutational screening; we could hypothesize that it would be useful for those patients where other markers are not available or who reached the MRD negativity.

**Electronic supplementary material:**

The online version of this article (10.1186/s12935-019-0807-0) contains supplementary material, which is available to authorized users.

## Background

Acute myeloid leukemia (AML), like other human malignant neoplasms, is a “dynamic” pathology, characterized by the acquisition of multiple somatic mutations, co-existing competing cellular clones, and clonal evolution over time. The individual and the genetic characteristics of the pathology represent the “classical” pre-treatment predictive and prognostic factors, but they are correct only in 75–80% of cases, indicating the importance of considering also further prognostic elements, such as the minimal/measurable residual disease (MRD) [1, 2].

Regarding the individual characteristics the most important parameters are represented by the performance status and age; in addition, the platelet count, the secondary nature of leukemia, the serum albumin and creatinine values, the leukocyte count and the percentage of blasts are other relevant variables.

According to the European Leukemia Network (ELN) classification, that includes also the cytogenetics, there are 3 classes of risk: favorable, intermediate and adverse. Compared to the previous version of guidelines edited in 2008, the ELN reccomandations state that, in the case of mutations of *NPM1* and of the biallelic aberrations of *CEBPA*, the coexistence of chromosomal alterations does not seem to modify their respective prognostic impact [3, 4]. Furthermore, the association of mutations of *NPM1* and *FLT3*-*ITD* with a low mutant/wild-type ratio (< 0.5), determines a similar (favorable) outcome, compared to that of patients carrying the mutation of *NPM1* but without *FLT3*-*ITD*; on the contrary, the prognosis worsens with a mutant/wild-type ratio > 0.5 [5]. Analogously, mutations in *RUNX1*, *ASXL1* and *TP53*, and the monosomal karyotype [6] have been now added to the adverse category.

The MRD is now considered as an independent prognostic indicator: it can be evaluated using cytofluorimetric or molecular tools: quantitative PCR is the molecular method generally preferred, but new technologies are today emerging, such as digital PCR (d-PCR) and next-generation sequencing (NGS). The MRD denotes the presence of leukemic cells with a sensitivity between 1:10^−4^ and 1:10^−6^, much greater than that offered by the morphological evaluation (5%), and by cytogenetic tools (1%). The sensitivity of flow cytometry is estimated to be at least 10^−4^ [7, 8], and also this method is today available from many laboratories. Nonetheless important differences between individual studies, an ever-growing body of data demonstrates that a positive MRD test at various time points identifies patients at particularly high risk of relapse and short survival, even after adjustment for other risk factors [9–11]. The currently used molecular markers are represented by *NPM1* mutations and rearrangements of *RUNX1*-*RUNXT1*, *CBFB*-*MYH11* and *PML*-*RARA*; for cases without these alterations, the flow cytometry is considered the best technique for investigating MRD.

The *NPM1* gene gives rise to nucleophosmin, a protein involved in DNA repair, apoptosis and regulation of the ARF-TP53 axis [12]. NPM1 shuttles normally between the nucleus and the cytoplasm; it is a molecular chaperone that prevents protein aggregation in the nucleolus and regulates the assembly and transport of preribosomal particles through the nuclear membrane [13, 14]. *NPM1* mutations result in traffic alterations and aberrant cytoplasmic dislocations of nucleophosmin. This unique immunohistochemical pattern led in 2005 to the discovery of *NPM1* mutations in AML [15]. Although more than 50 types of mutations have been detected in exon 12, 3 specific types (A, B and D) cover the 95% of all possible *NPM1* alterations [16]. The type A is the most common mutation, it is a duplication of a TCTG tetranucleotide at position 956 to 959 and accounts for up to 80% of cases. Mutations B and D are present in about 10% and 5% of *NPM1*-mutated AML [17]. The mutations of this gene characterize 30% of all forms of AML in adults [15], and reach a prevalence of 60% in cases with normal karyotype. *NPM1* mutations have been demonstrated to have an important prognostic value: in patients without cytogenetic alterations, without the co-presence of *FLT3*-*ITD*, they are associated with an overall survival (OS) longer than 5 years in 50–60% of cases [18, 19]. The same prognostic role this gene played also when used as marker of MRD, both on bone marrow and peripheral blood [20–23]. Literature shows conflicting data about relapsed *NPM1*-mutated AML; in 10% of cases, patients relapsed without *NPM1* mutations, so making confusing and debated the predictive role of this gene [20, 24, 25].

The “core-binding factor” (CBF) AMLs are characterized by specific chromosomal alterations: inv(16)(p13q22), t(16;16)(p13q22) leads to the *CBFB*-*MYH11* fusion gene, and t(8;21)(q22; q22) leads to the *RUNX1/RUNXT1*, formerly known as *AML1/ETO* fusion gene [26]. These fusion genes are found in 5–10% of the AMLs [27]; the prognosis of these forms is generally positive, with the achievement of complete remission (CR) in 80–95% of cases, and a 5-year OS of 48% [28]. In case of relapse (30% of cases), both fusion genes reoccur before the frank hematological relapse, which makes them good targets for monitoring MRD via quantitative PCR (QT-PCR) [29].

In addition to the markers recommended by the ENL guidelines, the wide use of *FLT3*-*ITD* and *WT1* in daily clinical practice must be taken also into consideration.

The duplications of internal segments of the *FLT3* gene (*FLT3*-*ITD*), coding for a tyrosine-kinase receptor, represent a common molecular alteration in AML, characterizing 25–30% of all cases [30]; it is associated with an adverse prognosis, which can however be mitigated in cases of treatment with FLT3 inhibitors, such as midostaurine [31]. *FLT3*-*ITD* is related to a poor prognosis, if the ratio between the expression of the mutant and the wild-type product is > 0.5 [32]. Point mutations in the tyrosine kinase domain of *FLT3* (*FLT3*-*TDK*) are instead observed in about 6% of AML, but their real prognostic significance is not yet demonstrated [33]. Mutated *FLT3*-*ITD* clones may appear or disappear during the history of disease: 6–33% of patients initially *FLT3*-*ITD*-mutated relapse without the mutation, whereas in 7–27% of cases who did not present it at the beginning, the mutation is present at the recurrence [34, 35].

The Wilm’s Tumor gene (*WT1)* is implicated in a series of malignant neoplasms, including the Wilms’ tumor, retinoblastoma, breast and lung cancer [36]. Its protein promotes cellular quiescence of hematopoietic stem cells, prevents apoptosis and induces differentiation [37]; in normal hematopoiesis, the expression of *WT1* is low and confined to the CD34+ cells [38]. In AML, 73–91% of cases hyper-express this gene, and 6–15% of the de novo forms have somatic mutations [39]. Studies aimed at evaluating the prognostic significance of *WT1* hyper-expression have led to conflicting results: some authors showed a reduced OS [40, 41], while other ones did not find a strong correlation between *WT1* expression levels and outcome [42]. Concerning the possibility of using *WT1* for assessing MRD, its continuous hyper-expression after induction would appear to make it a fairly specific indicator of relapse; at the time of transplantation, using a threshold of 100–250 copies/10^4^ of *ABL1* copies, the high gene expression seems to correlate with the risk of disease recurrence after transplantation, but the serial monitoring of this gene is not still recommended by the European guidelines.

About the additional mutations that can be detected in AML, the ELN consensus states that some mutational assays, such as those for *NPM1, CEBPA*, *RUNX1, FLT3, ASXL1,* and *TP53*, could be useful for predicting the outcome and helping to perform ab initio a patient-oriented therapy [1]. *ASXL1*, mutated in 5–30% of AML, regulates the chromatin remodeling, and seems to play a poor prognostic impact [43]; *TET2* is mutated in 7–23% of AMLs, but its predictive/prognostic role is still debated [44, 45]. *IDH1* and *IDH2* control the methylation status; found in 7–19% of AMLs, *IDH2* represents now an optimal target for enasidenib that offers 40% of responses to relapsed/refractory AML patients [46, 47]. *N*-*RAS* plays a relevant role in proliferation, differentiation, and apoptosis; its mutations occur in 7–17% of AML cases, with a poor prognostic impact [48, 49].

*c*-*KIT* mutations characterize 10–20% of the CBF AMLs [50], where they are associated with a higher risk of relapse [51]. *RUNX1* encodes for a transcription factor partner of numerous translocations, and seems to be correlated with a worse outcome [52]. *DNMT3A* is mutated in 20–30% of the AMLs, and its mutations represent a negative prognostic factor when combined with *FLT3* mutations [53–56].

Because of availability of the new techniques able to detect mutations with a higher sensitivity (NGS is able to detect mutations up to 1%), the use of mutations as predictive/prognostic tool has been today introduced in the clinical practice, as showed by the researchers at the Sanger Institute who elaborated an algorithm where clinical, cytogenetic, and molecular features are optimally integrated (available at the web site http://cancer.sanger.ac.uk/aml-multistage/).

The purpose of this retrospective study was to investigate in a series of 98 AML patients observed at the Hematology of Pisa (Italy) if detection of “additional” somatic mutations at baseline could represent an additional value in respect of the evaluation of the MRD post-induction/consolidation treatment in terms of event-free survival (EFS) and OS.

## Patients and methods

### Patients characteristics

This retrospective study enrolled 98 patients with AML observed at the Hematology Unit of the University of Pisa (Italy) between January 2015 and April 2018.

The characteristics of the enrolled subjects are shown in the Table 1; 57 patients were males and 41 females, with a median age at diagnosis of 58 years (range 19–89). In 9 cases the AML represented the evolution of a pre-existing myelodysplastic syndrome and in one case the leukemic evolution of a *JAK2*-mutated chronic myeloproliferative neoplasm. Based on the cytogenetic and molecular characteristics defined at diagnosis (ELN score), 25 patients were in the favorable risk group, 55 in the intermediate and 18 in the adverse one.

**Table 1:** Characteristics of patients

Number of patients	98
Age (median, range)	58 (19–89)
Sex	
M	57
F	41
Onset	
Primary	78 (80%)
Post-MDS/MPN	10 (10%)
Post-therapy	10 (10%)
Blasts % (median, range)	70 (20-95)
WBC, **(**median, range) x 10^9^/L	7.9 (0.8–35)
Hb, (median, range) x g/dL	9.6 (4–15.5)
PLT, **(**median, range) x 10^9^/L	50.5 (1.7–656)
Karyotype	
Normal	40 (41%)
Abnormal	43 (44%)
Complex	15 (15%)
WHO classification	
With recurrent abnormalities	44 (44%)
MDS-related	10 (10%)
Post-therapy	10 (10%)
Provisional entities	12 (12%)
NOS	19 (19%)
Myeloid sarcoma	3 (3%)
ELN risk	
Favorable	25 (25.5%)
Intermediate	55 (56.1%)
Adverse	18 (18.4%)
Treatment	
3 + 7	55 (56.1%)
Demethylating agents	13 (13.3%)
LAM 1310	17 (17.3%)
Supportive therapies	13 (13.3%)
AlloBMT	22 (22%)

### Treatment

About treatment, 55 cases (56.1%) received the “3 + 7” induction, based on anthracyclines (idarubicin 12 mg/m^2^/day, or daunorubicin 50 mg/m^2^/day, days 1–3, and aracytin 100 mg/m^2^/day, days 1–7), followed by two cycles of consolidation with aracytin 500 mg/m^2^ every 12 h, days 1–6, plus daunorubicin 50 mg/m^2^/day, days 4–6. Seventeen patients (17.3%) were enrolled in the GIMEMA LAM1310 protocol, so they received as induction cytarabine 100 mg/m^2^/day (days 1–10), etoposide 50 mg/m^2^/day (days 1–5), daunorubicin 50 mg/m^2^/day (days 1, 3, 5) followed by a consolidation with cytarabine 500 mg/m^2^ every 12 h (days 1–6) and daunorubicin 50 mg/m^2^/day (days 4, 5, 6).

Thirteen subjects (13.3%) were treated with demethylating agents, whereas 13 (13.3%) received only supportive therapies (etoposide, low-dose aracytin, fludarabine). In our series, 22 patients underwent to the myeloablative or reduced-intensity allogeneic transplant.

### Clinical response assessment

Complete remission (CR) was defined as bone marrow blasts < 5%, absence of circulating blasts or blasts with Auer rods, absence of extramedullary disease, ANC ≥ 1 × 10^9^/L, platelet count ≥ 100 × 10^9^/L. CR without minimal residual disease (CR^MRD−^) required CR plus negativity for a genetic marker by PCR, or flow cytometry. Partial remission (PR) was defined by all hematologic criteria of CR, decrease of bone marrow blast percentage to 5–25%, and decrease of pre-treatment bone marrow blast percentage by at least 50%.

Hematologic relapse required bone marrow blast percentage ≥ 5% or reappearance of blasts in the blood or development of extramedullary disease. Molecular relapse was defined as reoccurrence of pre-existing molecular markers when assessed by PCR or flow cytometry.

### Molecular analyses

Molecular analyses were performed at the time of diagnosis or just before the beginning of therapy, and repeated 30 days after induction and 30 days after consolidation. All cases were censored at the allogeneic transplantation time-point; during the follow-up, patients were followed for MRD every 3 months for 2–3 years, according to the medical decision.

At baseline, all samples were tested for *BCR/ABL1*, *AML1/ETO*, *inv(16)*, *NPM1* mutations, *FLT3* mutations (*FLT3*-*ITD* and *TDK*), and *WT1* expression. In addition, mutations of 11 genes already considered as significant in AML were assessed by PCR (see in the following).

Patients suffering from promyelocytic leukemia, which is a separate entity, were excluded from the study.

### Nucleic acids extraction

The DNA extraction from 12 mL of bone marrow/peripheral blood anti-coagulated with EDTA was performed using the automatic apparatus BioRobot EZ1, theEZ1 DNA Blood Card, and the EZ1 DNA Blood 350 µL Kit (Qiagen^®^, Valencia, CA, USA).

RNA extraction was performed using automatic apparatus Maxwell16 Promega and the Maxwell^®^ RSC simply RNA Blood Kit (Promega^®^).

The extracted nucleic acids was quantitated using the Thermo Scientific Nano Drop 2000 spectrophotometer (Thermo Fisher Scientific^®^, Wilmington, DE, USA).

### Quantitative analysis of NPM1 and WT1 expression

The expression of *NPM1* was evaluated by Ipsogen^®^ *NPM1* mutA MutaQuant^®^ Kit, and Ipsogen^®^ *NPM1* mutB&D MutaQuant^®^ Kit. Quantitative PCR for *WT1* expression was performed with the Ipsogen^®^ *WT1* ProfileQuant^®^ Kit. The sensitivities specified by the manufacturer were 0.01%. Both analyses were performed on CFX Connect^®^ (Bio-Rad).

### Qualitative analysis for FLT3 ITD mutations

Qualitative PCR for *FLT3*-*ITD* mutations was performed as already described [30, 57]; sensitivity was 0.5% and PCR products were analyzed by capillary electrophoresis.

### Detection of somatic mutations by PCR

Mutation detection tests were performed by using the qBiomarker Somatic Mutation PCR Arrays (Qiagen, Milan, Italy); each array included hot spot mutations for *ASXL1*, *TET2*, *IDH1*, *IDH2*, *N*-*RAS*, *WT1*, *c*-*KIT*, *RUNX1*, *DNMT3A*, *FLT3*, and *NPM1*, for a total of 83 possible mutation sites (see Table 2). Each analysis was performed by using the Amplification Refractory Mutation System (ARMS) technology and results calculated by the ΔΔCt method. Samples were considered as wild-type if the ΔΔCt was < 3; border-line for ΔΔCt between 3 and 4, and mutated if the ΔΔCt was > 4.

**Table 2:** Detailed structure of the mutational PCR plate

Gene	Nucleotide replacement	Aminoacid replacement
ASXL1	c.1772_1773insA	p.Y591fs*1
ASXL1	c.1888_1909del22	p.H630fs*66
ASXL1	c.2302C > T	p.Q768*
ASXL1	c.2324T > G	p.L775*
ASXL1	c.3202C > T	p.R1068*
DNMT3A	c.2644C > T	p.R882C
DNMT3A	c.2711C > T	p.P904L
FLT3	c.1803_1804ins	p.L601_K602ins27
FLT3	c.2503G > C	p.D835H
FLT3	c.2503G > T	p.D835Y
FLT3	c.2505T > G	p.D835E
FLT3	c.2508_2510delCAT	p.I836del
IDH1	c.394C > A	p.R132S
IDH1	c.394C > G	p.R132G
IDH1	c.394C > T	p.R132C
IDH1	c.395G > A	p.R132H
IDH1	c.395G > T	p.R132L
IDH2	c.418C > T	p.R140W
IDH2	c.419G > A	p.R140Q
IDH2	c.419G > T	p.R140L
IDH2	c.514A > T	p.R172W
IDH2	c.515G > A	p.R172K
IDH2	c.515G > T	p.R172M
IDH2	c.516G > T	p.R172S
KIT	c.1509_1510insGCCTAT	p.Y503_F504insAY
KIT	c.1621A > C	p.M541L
KIT	c.1656_1673del18	p.Y553_K558>
KIT	c.1667_1672delAGTGGA	p.W557_K558del
KIT	c.1669_1683del15	p.W557_E561del
KIT	c.1669T > A	p.W557R
KIT	c.1669T > C	p.W557R
KIT	c.1670_1675delGGAAGG	p.W557_V559 > F
KIT	c.1675_1677delGTT	p.V559del
KIT	c.1676T > A	p.V559D
KIT	c.1676T > C	p.V559A
KIT	c.1676T > G	p.V559G
KIT	c.1679T > A	p.V560D
KIT	c.1708_1728del21	p.Y570_L576del
KIT	c.1735_1737delGAT	p.D579del
KIT	c.1924A > G	p.K642E
KIT	c.1961T > C	p.V654A
KIT	c.2446G > C	p.D816H
KIT	c.2446G > T	p.D816Y
KIT	c.2447A > T	p.D816V
KIT	c.2466T > A	p.N822K
KIT	c.2466T > G	p.N822K
KIT	c.2467T > G	p.Y823D
KIT	c.2474T > C	p.V825A
NPM1	c.863_864insCATG	p.W288fs*12
NPM1	c.863_864insCCGG	p.W288fs*12
NPM1	c.863_864insCCTG	p.W288fs*12
NPM1	c.863_864insTATG	p.W288fs*12
NPM1	c.863_864insTCTG	p.W288fs*12
NRAS	c.181C > A	p.Q61K
NRAS	c.182A > C	p.Q61P
NRAS	c.182A > G	p.Q61R
NRAS	c.182A > T	p.Q61L
NRAS	c.183A > C	p.Q61H
NRAS	c.183A > T	p.Q61H
NRAS	c.34G > A	p.G12S
NRAS	c.34G > T	p.G12C
NRAS	c.35G > A	p.G12D
NRAS	c.35G > C	p.G12A
NRAS	c.35G > T	p.G12V
NRAS	c.37G > C	p.G13R
NRAS	c.37G > T	p.G13C
NRAS	c.38G > A	p.G13D
NRAS	c.38G > C	p.G13A
NRAS	c.38G > T	p.G13V
NRAS	c.52G > A	p.A18T
RUNX1	c.167T > C	p.L56S
RUNX1	c.319C > T	p.R107C
RUNX1	c.496C > T	p.R166*
RUNX1	c.592G > A	p.D198 N
RUNX1	c.593A > G	p.D198G
RUNX1	c.602G > A	p.R201Q
RUNX1	c.611G > A	p.R204Q
TET2	c.1648C > T	p.R550*
TET2	c.2746C > T	p.Q916*
WT1	c.1168C > T	p.R390*
WT1	c.906_907insT	p.V303fs*14
WT1	c.938C > A	p.S313*
WT1	c.940_941insTCGG	p.A314fs*4

### Statistical analysis

All statistical analyses were performed by using the SPSS 22.0 software (SPSS Inc, Bologna, Italy). OS was calculated from the date of diagnosis to death or last follow-up; EFS was measured from the start of the induction to the last follow-up, disease progression, definitive discontinuation of treatment or relapse. Survival curves were calculated using the Kaplan–Meier method, censoring cases at the date of the eventual bone marrow transplantation. The Log-rank method was adopted for comparison of survivals between two groups.

The clinical responses have been evaluated either after induction or after consolidation; nevertheless, only those observed after induction have been used for the statistical analyses.

The Chi squared test, Fisher’s exact test, and Kruskal–Wallis’s test were used to compare variables when appropriate. All statistical comparisons were two-sided.

## Results

### Diagnosis: the mutational landscape

At baselines, 98 cases were tested for *NPM1* and *FLT3* mutations, and 31 patients were also assessed for 83 different hot spot mutations belonging to 11 different genes (see Methods): 59/98 patients (60.2%) resulted mutated, for overall 106 mutations detected: 31 cases carried *FLT3* mutations (31.6%), of them 24 (24.5%) were *FLT3*-*ITD* and 5 (5.1%) were *FLT3*-*TDK*; 38 patients resulted NPM1-mutated (38.7%), and 11 subjects, who were NPM1- and FLT3 wild-type, presented additional mutations (11.2%). The overall prevalence of mutations in our series is reported in the Table 3 and in more detail in the Heat Map (see Additional file 1).

**Table 3:** Prevalence of all mutation

Mutation	% in whole series (%)
Overall mutational rate	60.2
NPM1	38.7
FLT3-ITD	24.5
c-KIT	48.4
N-RAS	25.8
IDH1	9.6
FLT3-TDK	5.1
IDH2	19.3
DNMT3A	9.6
WT1	12.9
RUNX1	9.6
TET2	0
ASXL1	0
WT1 high (expression)	84.6

In the 38 cases with *NPM1* mutations, 29 (76.3%) were type A, 3 (7.9%) were type B and 6 (15.8%) were type D. Regarding ELN risk category, 25 (65.8%) were in the ”favorable” group, 12 (31.6%) were in the “intermediate”, and 1 (2.6%) case presented an “adverse” score, due to the finding of a complex karyotype (p < 0.01). The quantitative PCR showed at baseline a *NPM1* mean mutational burden of 380 (range 0.03–940).

In subjects with *FLT3*-*ITD* mutations, 5 (20.8%) were included in the ”favorable” ELN category, 14 (58.4%) in the “intermediate”, and 5 (20.8%) in the “adverse” one. In 16 out of 24 cases (66.7%), the mutant/wild-type allelic ratio was > 0.5; at baseline, the median allelic ratio was 0.72 (range 0.01–4.4).

Overall 71% of our patients showed at least an additional mutation: the most common are resulted *c*-*KIT*, with 15 out of the 31 tested patients (48.4%) showing these DNA abnormalities, followed by *N*-*RAS*, mutated in 8 cases (25.8%). Moreover, 6 cases (19.3%) carried mutations of *IDH2*, and 3 patients (9.6%) of *IDH1*; 3 patients were mutated for *RUNX1* (9.6%), 4 cases (12.9%) for *WT1*, while *DNMT3A* mutations were found in 3 cases (9.6%). No patients presented *TET2* or *ASXL1* mutations. Overall, 34.7% of all patients presented > 1 mutation; no clustered associations were observed in our series.

Concerning *WT1*, patients were distinguished based on its expression level in *WT1*^high^ and *WT1*^low^, considering as cut-off values those identified by the manufacturer (50/10^−4^ ABL1 copies for PB and 250/10^−4^ ABL1 copies for BM) [58]. Eighty-three of our patients (84.6%) showed a high *WT1* expression, with values ranging from 499 to 18,885, and a mean of 6145. *WT1* expression levels were not significantly different according to sex, age, WHO classification, or cytogenetic score.

### Predictive and prognostic value of the clinical and genomic features at diagnosis

At the end of the induction, 77 of the initial 98 cases were evaluable; the 21 cases lost to follow-up were those who came back to the near local centers that usually refer patients to Pisa for the initial centralized diagnosis and therapeutic decisions.

After induction, 60% of our patients achieved a CR, and 12.3% a PR, for an overall response rate (ORR) of 72.3%; after consolidation, the CR rate increased to 76.3%, and the PR rate to 15.8%, for an ORR of 92.1%. When CR rates where compared between the classic “3 + 7” and the GIMEMA LAM 1310 trial, no differences were observed.

We evaluated the predictive value (in terms of quality of response) of the clinical and genetic features: gender (female versus male), age (cut off 65 years), ELN risk score, presence or absence of a complex karyotype, white blood cells count (WBC), platelets count (PLT), hemoglobin values (Hb), number of blasts, mutational landscape (*FLT3*, *NPM1*, additional mutations and *WT1* expression levels).

We found that the age at diagnosis < 65 years and a “favorable” ELN score were the parameters capable of significantly predicting the attainment of CR. In particular, 85% of subjects younger than 65 years achieved CR versus 46% of patients over 65 (p = 0.002); on the other hand, in the group of non-responsive patients, only 11% were in the “favorable” ELN risk category, while 42% and 46% were at “intermediate” and “adverse” risk, respectively (p = 0.042).

As concerns the molecular variables, a significant correlation emerged between *NPM1* status and the probability of obtaining CR: as expected, 78% of the *NPM1*-mutated patients obtained an excellent response compared to 42% of *NPM1*-wild type subjects (p = 0.008). When CR rate were compared between the two induction regimens (“3 + 7” vs GIMEMA LAM 1310), the impact of the NPM1 mutations on the CR achievement still remained significant only in the 3 + 7 cohort (p = 0.006).

The presence of *FLT3* mutations did not significantly correlate with CR; however, even in this case, the initial mutational burden was predictive of the response, with mutated/wild-type allele ratio of 1.37 ± 0.44 in failing cases versus 0.67 ± 0.55 of cases achieving CR (p = 0.034).

Finally, also the presence of additional mutations represented a poor predictive factor: indeed, only 19% of cases with additional mutations achieved CR after induction in comparison to 43% of the subjects without any somatic mutations, independently from the presence of *NPM1* or *FLT3* mutations (p = 0.041).

On the contrary, basal *WT1* expression levels did not significantly impact on the CR achievement.

During follow-up, 45 out of the 77 evaluable cases (58.4%) died, 56 (72.7%) relapsed and underwent to a re-induction treatment with the re-obtainment of response in half of the cases, mostly after the FLANG regimen (association of fludarabine, cytarabine and mitoxantrone).

Concerning the prognostic value, we assessed firstly if the clinical and mutational characteristics found at baseline could significantly affect OS and EFS of our patients.

**Fig. 1:**
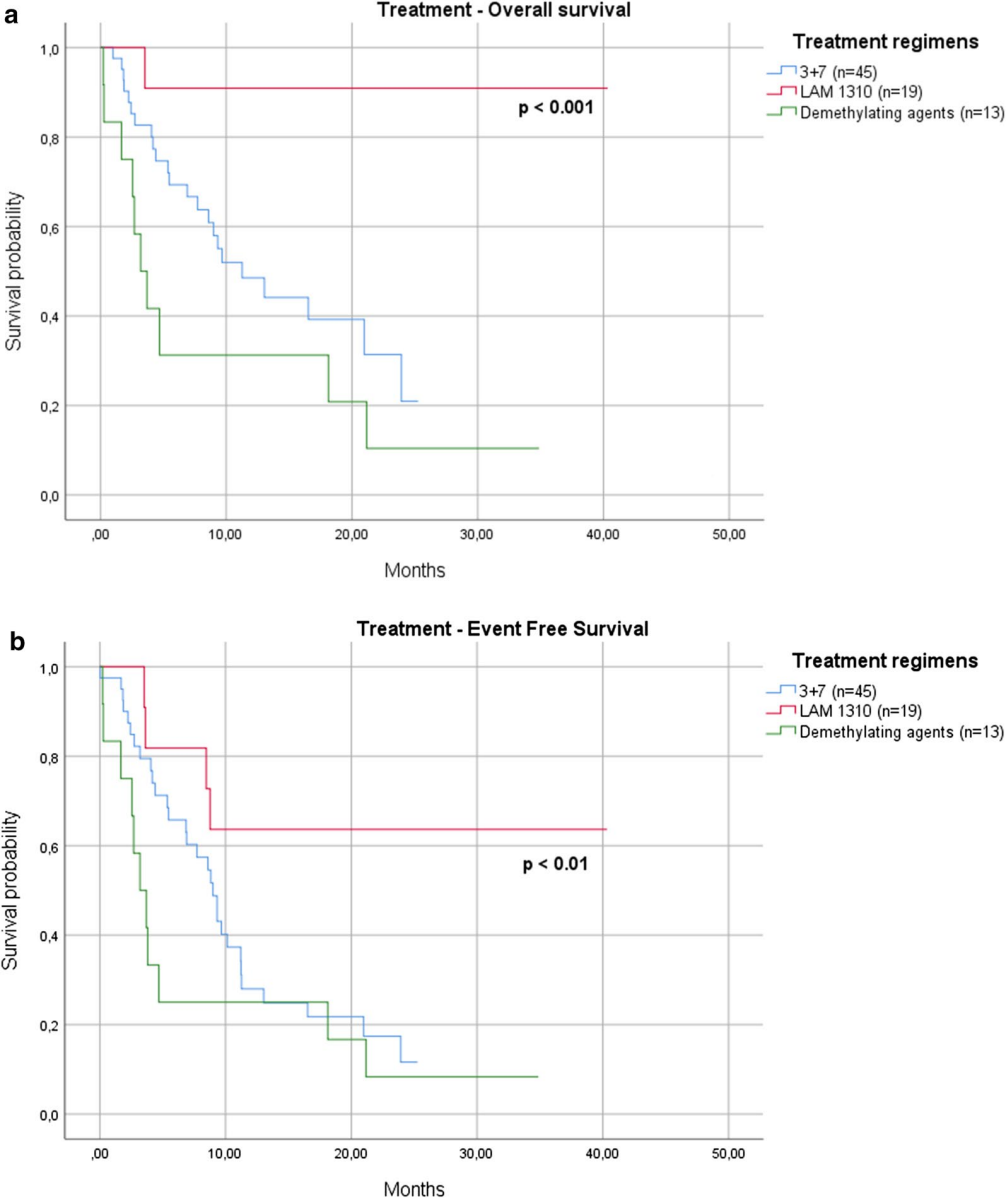
Therapeutic regimens. No differences in terms of length of survival have been observed between cases treated with demethylating agents in comparison to 3 + 7 regimen (**a**). EFS was 32% at 12 months, and 19% at 24 months in the whole series; even in this case, patients enrolled in the GIMEMA trial showed a clear advantage, with 24 months-EFS of 65% versus 12% of the subjects receiving 3 + 7 and 8% of those receiving demethylating drugs (p < 0.001) (**b**)

In the whole series, the 12- and 24-months OS were 43% and 30%, respectively; 24-months OS reached 90% in cases enrolled in the GIMEMA AML 1310 trial (see “Patients and methods” section), probably because in that study younger cases with a better performance status were enrolled (p < 0.001). No differences in terms of length of survival have been observed between cases treated with demethylating agents in comparison to 3 + 7 regimen (anthracyclines and aracytin) (Fig. 1a). EFS was 32% at 12 months, and 19% at 24 months in the whole series; even in this case, patients enrolled in the GIMEMA trial showed a clear advantage, with 24 months-EFS of 65% versus 12% of the subjects receiving 3 + 7 and 8% of those receiving demethylating drugs (p < 0.01) (Fig. 1b).

**Fig. 2:**
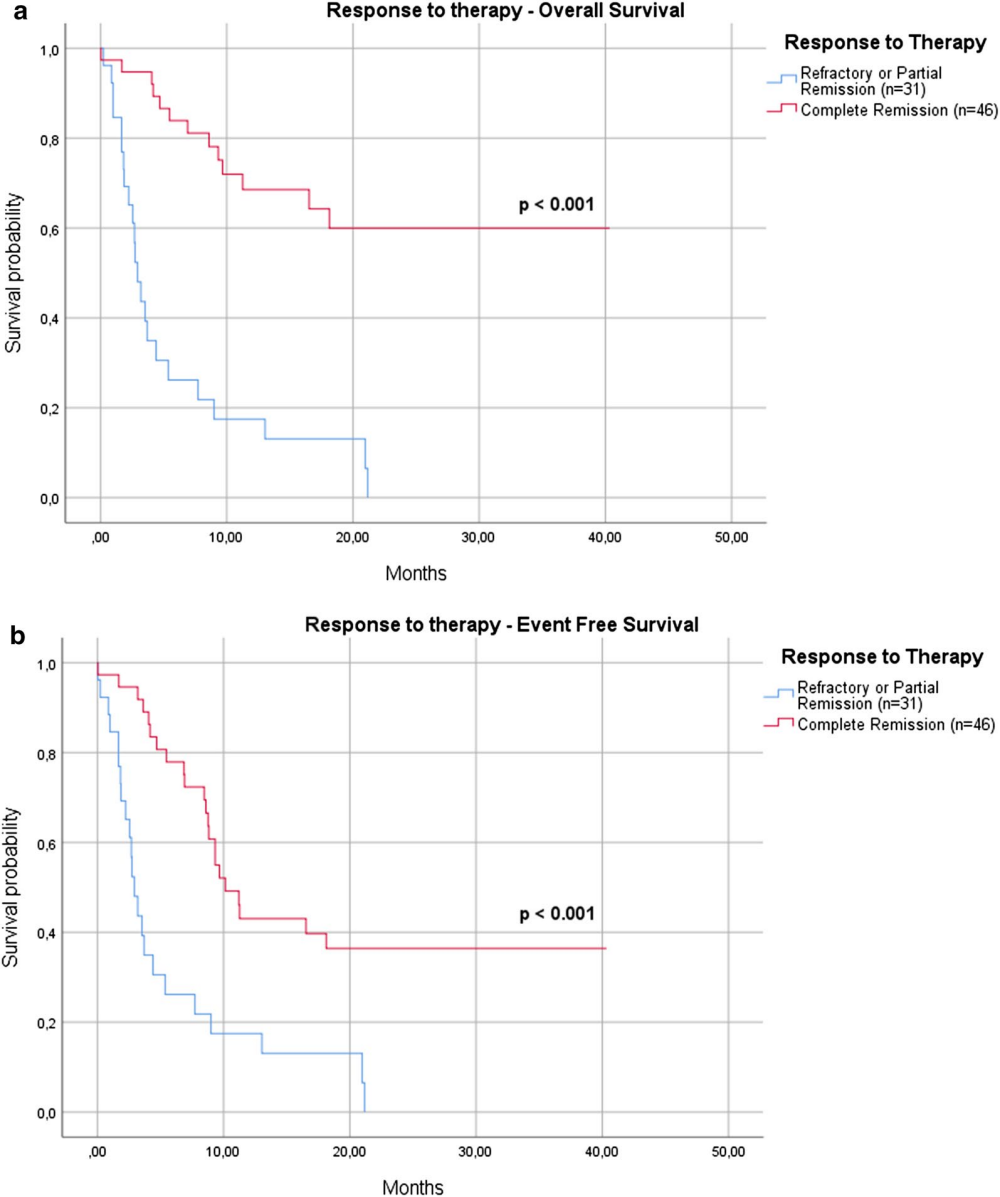
Response to induction therapy. The OS was significantly conditioned by the response to therapy, with the median not reached at 12 months by patients in CR compared to only 3 months for those who achieved only a PR or did not respond to treatment (p < 0.001) (**a**). Also EFS was significantly conditioned, with 43% of patients in CR who were free from events at 12-months versus 18% of those who did not reach a complete response (p < 0.001) (**b**)

Regarding the clinical features, OS was significantly higher for younger subjects, with a median OS of 20 months for patients < 65 years versus 4 months for those > 65 years (p < 0.001). The OS was significantly conditioned by the response to induction therapy, with the median not reached at 12 months by patients in CR compared to only 3 months for those who achieved only a PR or did not respond to treatment (p < 0.001) (Fig. 2a). Also EFS was significantly conditioned by the response to therapy, with 43% of patients in CR who were free from events at 12-months versus 18% of those who did not reach a complete response (p < 0.001) (Fig. 2b).

**Fig. 3:**
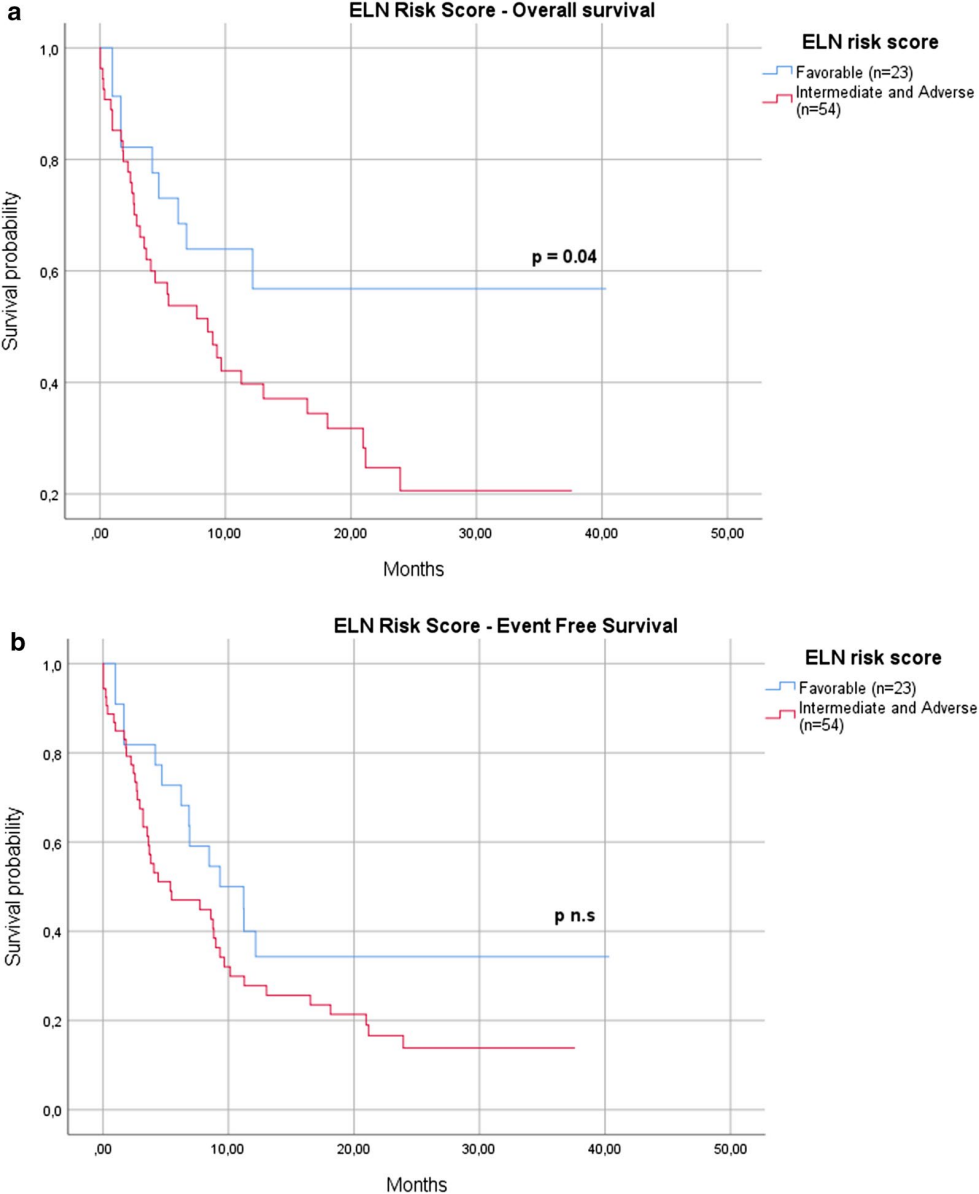
ELN risk score. OS (**a**), but not EFS (**b**), was significantly conditioned by the ELN risk score, with 57% of patients living at 12 months in the subgroup of those at “favorable” risk versus 40% of those at “intermediate/adverse risk” (p = 0.04)

Moreover, OS, but not EFS, was significantly conditioned by the ELN risk score, with 57% of patients living at 12 months in the subgroup of those at “favorable” risk versus 40% of those at “intermediate/adverse risk” (p = 0.04) (Fig. 3a, b).

Concerning the mutational landscape, none of the molecular parameters analyzed at baseline (mutation of *NPM1*, *FLT3*-*ITD* including their mutational burden, over-expression of *WT1*, presence of somatic mutations) resulted to significantly condition OS or EFS.

### MRD: FLT3, NPM1 and WT1

After induction, 39% of the initially *NPM1*-mutated cases became MRD-negative; in the still positive subjects, the mutational burden reduced from a mean value of 380 to 35 (reduction > 1 log).

On the other end, 60% of the *FLT3*-mutated cases became negative; differently from the *NPM1*, for this marker no significant quantitative changes were measured.

Finally, in the subgroup of patients with concomitant *NPM1* and *FLT3* mutations, 60% of patients achieved the MRD negativity; interestingly, in 12% of them a different behavior between *FLT3* and *NPM1* was observed (cases already *FLT3*-*ITD*-negative sometimes still remaining *NPM1*-positive). This phenomenon could be probably related to a different sensitivity of the used molecular techniques (10^−4^ for *NPM1* and 10^−2^ for *FLT3*-*ITD*).

After induction, 55% of *WT1*^high^ cases reduced their molecular burden, whereas 25% of *WT1*^low^ patients increased the gene expression levels; nevertheless, therapy allowed the mutational burden to reduce from 4575/10^−4^ to 93.50/10^−4^.

Overall, after the initial treatment, the number of patients carrying mutations decreased from 74 to 57.4%.

### Predictive and prognostic value of MRD

In the MRD predictive and prognostic evaluation all patients who achieved CR (46) were included.

When we assessed the prognostic role of MRD, we found that: 1) the entity of clearance of *NPM1* mutational load significantly conditioned both OS and EFS, with 100% of subjects being alive at 12 months in the cohort of those showing a reduction > 1 log versus 44% of those with a reduction < 1 log (p = 0.05). Analogously, the 12-months EFS was 71% for patients with a reduction > 1 log versus 0% of those with a reduction < 1 log (p = 0.005). The one log reduction has been chosen as a cut-off as it represented the average reduction of mutational burden of our patients between diagnosis and post-induction. It has to be considered that, at the opposite, the qualitative status of *NPM1* (mutated or wild-type) after induction treatment did not significantly impact on survival.

**Fig. 4:**
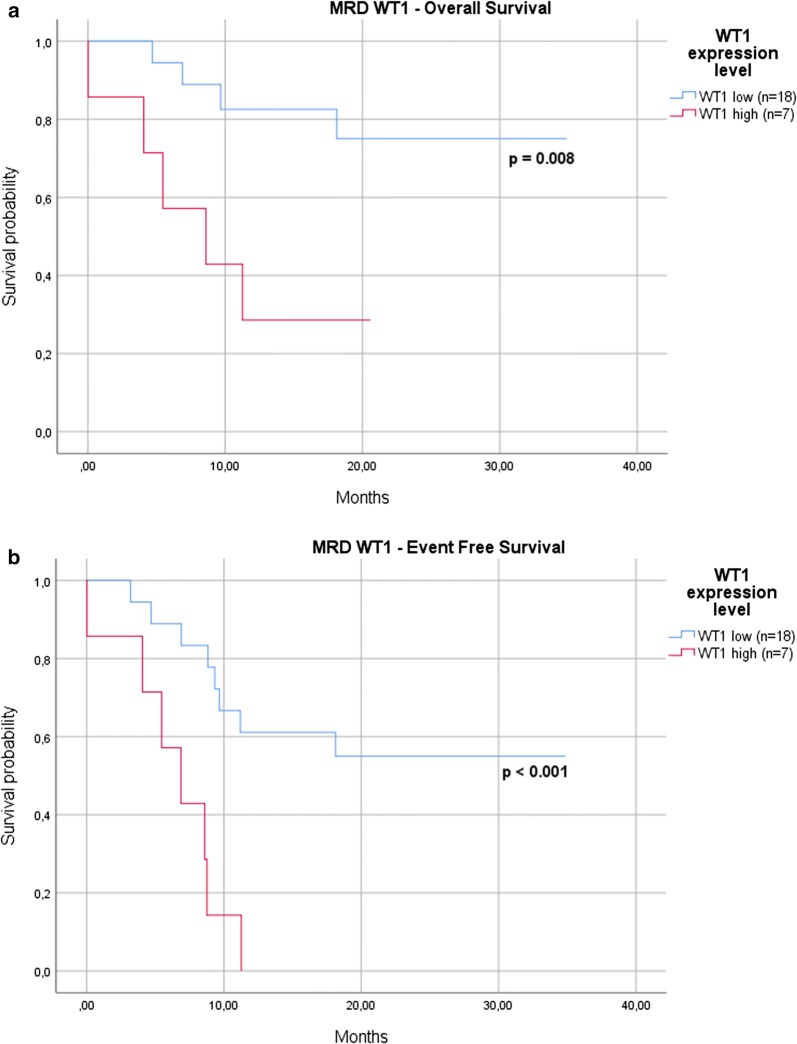
WT1 expression levels. The *WT1* expression levels significantly impacted on survival: the 12-months OS was 83% for cases with low levels after induction versus 29% for those with high *WT1* expression levels (p = 0.008) (**a**), and EFS was 0% for cases with high versus 61% for those with low *WT1* expression (p < 0.001) (**b**)

2) the clearance of *FLT3* played a favorable impact on both OS and EFS: indeed, the median OS was 4 months for still *FLT3*-mutated cases versus not reached in the group of patients who became *FLT3*-negative (p = 0.008), and EFS was 0% for MRD-positive cases versus 73% for the MRD-negative ones (p = 0.004).

3) the *WT1* expression levels significantly impacted on survival: the 12-months OS was 83% for cases with low levels after induction versus 29% for those with high *WT1* expression levels (p = 0.008) (Fig. 4a), and EFS was 0% for cases with high versus 61% for those with low *WT1* expression (p < 0.001) (Fig. 4b).

4) about somatic mutations, nor OS or EFS were significantly conditioned by the presence/absence of additional mutations at baseline.

Then, we combined data becoming from the MRD status with the presence/absence of some somatic mutations at baseline in order to test if these mutations could represent an adjunctive value to the assessment of MRD, so to justify a further molecular analysis in all our patients at diagnosis. No differences were observed in terms of EFS; concerning OS, we observed that in the subgroup of MRD-negative cases the presence of additional mutations at baseline impaired OS of 20%, even if this value did not reach the statistical significance probably because of the small number of patients enrolled.

In conclusion, even if the somatic mutations revealed to have a significant negative predictive power in terms of quality of response to the induction, their prognostic role remains a matter of debate.

Regarding the possibility of predicting relapse/progression by *NPM1*, *FLT3*-*ITD* or *WT1* behavior, we performed longitudinal molecular assays during follow-up in 26 cases.

Among patients with *NPM1* mutations at diagnosis, 22% relapsed: when we backtracked the molecular status of this gene, we observed in half of cases an increase of the mutational burden at the time-point immediately preceding the hematological relapse (about 2 months earlier).

In the subgroup of the *FLT3*-mutated patients, neither the persistence of mutations nor their ratio showed any “anticipatory” predictive power.

Concerning *WT1*, an increase of its expression levels was able to anticipate disease recurrence in 7/11 relapsed cases (64%).

Overall, even if a small series of cases, we would suggest that the quantitative monitoring of *NPM1* and *WT1* could be worth of being performed in the routine clinical practice.

## Discussion

The use in the clinical practice of MRD evaluation for predicting response to the induction treatment or long-term prognosis is still a hot topic for discussion, given the lack of variables that represent certain powerful factors, even if the use of advanced cytofluorimetric and molecular techniques in the definition of MRD largely improved the sensitivity deriving only from the morphological evaluations.

In this work, even if with the limit of a retrospective and small study, we reviewed the histories of 98 patients affected by AML afferent to our Hematology Unit from 2015 to 2018, focusing mainly on the identification of factors eventually able to predict response and survival.

In particular, we wondered if: (1) the evaluation of molecular MRD could have a real prognostic value; (2) which of the possible candidate genes among *FLT3*-*ITD*, *NPM1*, and *WT1* would reveal the highest prognostic significance, but especially (3) if the assessment of some additional somatic mutations at diagnosis could represent an adjunctive prognostic value in respect to the evaluation of MRD alone.

Obviously, we have firstly to consider that ELN suggests the monitoring of MRD as part of the standards of care for AML patients and that the recommended molecular markers are the core-binding factors translocations, the *NPM1* mutations and, in acute promyelocytic leukemia, the *PML*-*RARA* translocation.

According to the ELN suggestions, *WT1* expression should not be used, except in cases that do not show other “validated” molecular markers, because of the low sensitivity and specificity of this gene.

Finally, also monitoring of *FLT3*-*ITD* mutations is not yet recommended, because mutations of this gene may occur at relapse with several additional gene segments or deletions different from those observed at diagnosis [2].

Concerning the molecular markers, our attention was mainly focused on three genes already commonly used in the clinical practice: mutations of *NPM1* and *FLT3*-*ITD*, and *WT1* expression levels.

Regarding *NPM1*, our study, in accordance with the ELN recommendations and literature [23, 59], confirmed its important prognostic role, reaching the conclusion that the rapid reduction of its mutational burden after induction (> 1 log) would be a potential valid predictive/prognostic tool. In addition, this observation well fits with what emerged in the context of other hematological diseases, such as in chronic myeloid leukemia, where the early and rapid reduction of the *BCR*-*ABL1* transcript during treatment with tyrosine kinase inhibitors is associated with a greater probability of obtaining the deep molecular response and prolonged OS [60].

Contrary to what suggested by ELN, our study also attributed a significant prognostic value to the *WT1* expression and to the *FLT3*-*ITD* mutations. We observed that MRD negativity for *FLT3*-*ITD* were obtained earlier than that for *NPM1*, although no specific *FLT3* inhibitors were used in combination with the induction therapy. This observation could be probably linked to the different sensitivity of methods used for molecular analyses: in fact, the sensitivity of the tests for *NPM1* resulted, in our hands, more than one logarithm higher than that of PCR used for assessing the *FLT3* mutations.

As further novelty, we assessed by a simple PCR methods the presence of 83 possible hot spot mutations (in 11 genes) at baseline, in order to initially better characterize our patients. Of course we analyzed only few and arbitrarily chosen genes, but we supposed that this additional mutational load (selected among the most relevant genes in AML) could play an important prognostic/predictive role. Indeed, we already demonstrated that the addition of these mutational evaluations to the Sanger’s algorithm (see Introduction) impaired the prognosis of more than half of cases (data submitted). Moreover, the technical novelty was also the employ of PCR instead of NGS for detecting mutations, with an evident and practical advantage also for small laboratories where NGS is not still suitable. In our series the additional mutations clearly played a negative impact on the achievement of a satisfying response to the induction. On the contrary, they did not achieve the statistical significance in terms of OS or EFS probably because the small number of patients tested in this study (31), nevertheless the presence of these mutations impaired the prognosis in the 20% of cases defined as MRD-negative. Obviously, further and larger prospective studies will be necessary for definitively understand if the assessment of these mutations could be really cost-effective in the daily routine.

## Conclusions

In conclusion, our results, even if coming from a quite small and retrospective study, are substantially in line with the international recommendations. In addition to data already published, we demonstrated that additional somatic mutations, present in a large number of cases, are able to impair outcome of the already MRD-negative subjects. About MRD, our results suggest a prognostic role also for the *WT1* expression. Finally we considered as relevant the assessment of *NPM1* quantity clearance instead of the presence/absence of mutations alone.

Still remains in doubt the utility in terms of long-term prognosis of a baseline more complex and large mutational screening (perhaps performed by NGS); on the basis of our results, we could hypothesize that it would be useful for those patients where other markers (such as *WT1*, *FLT3*-*ITD*, and *NPM1*) are not available. Nevertheless, we have to not forget that a good characterization of our AML patients is today more and more necessary for leading ab initio the choice of the most appropriate treatment, in the new era where target therapies, such as those against FLT3, c-KIT, IDH1 or IDH2 are a reality.

## Additional file

**Additional file 1.** Mutation prevalence heat map. Graphical representation of the overall prevalence of mutations in each patient of our series at baseline. Mutated in red, wild-type in green, yellow for normal expression level, orange for higher gene expression.

## Abbreviations

AML: acute myeloid leukemia
EFS: event free survival
OS: overall survival
MRD: minimal/measurable residual disease
ELN: European Leukemia Network
NGS: next-generation sequencing
CBF: core-binding factor
CR: complete remission
CR^MRD−^: complete remission without minimal/measurable residual disease
PR: partial remission
ORR: overall response rate
WBC: white blood count
PLT: platelets count
Hb: hemoglobin values
